# Large sharing networks and unusual injection practices explain the rapid rise in HIV among IDUs in Sargodha, Pakistan

**DOI:** 10.1186/1477-7517-6-13

**Published:** 2009-06-26

**Authors:** Adnan A Khan, Ahmad B Awan, Salman U Qureshi, Ali Razaque, Syed T Zafar

**Affiliations:** 1 Research and Development Solutions, Islamabad, Pakistan; 2 National AIDS Control Programme, The Ministry of Health, Islamabad, Pakistan; 3 Nai Zindagi Trust, House 935, Block J-2, Blue Area, Johar Town, Islamabad, Pakistan; 4 Punjab AIDS Control Program, Lahore, Pakistan

## Abstract

**Background:**

Of the nearly 100,000 street-based IDUs in Pakistan, 20% have HIV. We investigated the recent rise in HIV prevalence from 12 to 52% among IDUs in Sargodha despite > 70% coverage with syringe exchanges.

**Methods:**

We interviewed approximately 150 IDUs and 30 outreach workers in focus group discussions.

**Results:**

We found six rural and 28 urban injecting locations. Urban locations have about 20–30 people at any time and about 100 daily; rural locations have twice as many (national average: 4–15). About half of the IDUs started injecting within the past 2 years and are not proficient at injecting themselves. They use street injectors, who have 15–16 clients daily. Heroin is almost exclusively the drug used. Most inject 5–7 times daily.

Nearly all injectors claim to use fresh syringes. However, they load, inject and share using a locally developed method called scale. Most Pakistani IDUs prefer to double pump drug the syringe, which allows mixing of blood with drug in the syringe. The injector injects 3 ml and keeps 2 ml (the scale) as injection fee. The injector usually pools all the leftover scale (now with some blood mixed with drug) either for his own use or to sell it. Most IDUs backload the scale they buy into their own fresh syringes.

**Discussion:**

Use of an unprecedented method of injecting drugs that largely bypasses fresh syringes, larger size of sharing networks, higher injection frequency and near universal use of street injectors likely explain for the rapid rise in HIV prevalence among IDUs in Sargodha despite high level provision of fresh syringes. This had been missed by us and the national surveillance, which is quantitative. We have addressed this by hiring injectors as peer outreach workers and increasing syringe supply. Our findings highlight both the importance of qualitative research and operations research to enrich the quality of HIV prevention programs.

## Background

Ever since an outbreak heralded its onset in 2003 [1-3], the HIV epidemic has expanded explosively among Pakistan's injection drug users (IDUs). The National AIDS Control Program (2006) estimates that there are an estimated 146,000 IDUs in Pakistan[4] of which about 80,000 to 100,000 are street-based. By 2007, nearly 20% of these street-based IDUs nationwide were infected with HIV[5]. Most inject about 2–3 times a day and in groups of 4–10. Specific drugs used vary by city and include opioids (e.g. heroin, buprenorphine), benzodiazepines (e.g. diazepam), solvents (e.g. rubber glue), antihistamines (e.g. Phenirimine) etc[5-8]. Overall the experience in Pakistan has been consistent with that from other Asian countries where HIV epidemics were started among IDUs as well[9,10].

The mainstay of HIV prevention for IDUs is syringe-needle exchange [11-16]. Behavior change counseling, detoxification, rehabilitation, social and medical services, antiretroviral therapy and oral substitution therapy also play a vital role [17-22]. All these interventions are available in Pakistan except oral substitution therapy. They are offered as part of citywide projects that are funded by the government and implemented by non-governmental organizations. Nai Zindagi (New Life) is one such organization that implements HIV interventions for about 12,000 IDUs in four cities including Sargodha. The interventions provided include syringe-needle exchange, voluntary counseling and testing (VCT) for HIV, wound and healthcare, counseling, detoxification and rehabilitation.

In December 2006, the national surveillance showed that HIV prevalence in Sargodha had reached 51.3%[5]; up from 12% in a survey from July 2005[8]. These latter results were consistent with Nai Zindagi's VCT data. The same survey also showed that there were about 2450 street-based IDUs in Sargodha, who were a median of 30 years old and had injected for about 3 years. Over 70% live at home with family or relatives. Most (75%) report using street injectors some or most of the time and 38% had done so with their last injection. They injected a median of 3 times a day and over 80% reported injecting their last dose in a group. Heroin was the principal drug used. However, 90% reported using a new syringe with their last injection and of the remaining, 88% reported cleaning their syringes (usually with water)[5]. In the 18 months prior to December 2006, the NGO had registered 1450 clients and supplied 390,000 syringes (but not any other injecting paraphernalia). There are no other services available for IDUs in the city.

Experience from Vancouver, B.C., Canada has shown that mere syringe distribution is insufficient for curtailing HIV transmission. While many factors limited the efficacy of the program (users transitioning to cocaine, more frequent users availing the services etc), a key problem seen in Vancouver was that the injecting behaviors of IDUs changed insufficiently despite the availability of new syringes[23]. This was not the case in Sargodha where nearly all IDUs reported either using new syringes for injections or cleaning their old syringes before use. We conducted this assessment to understand why HIV had spread so explosively among IDUs in Sargodha, despite a consistent supply of new syringes to approximately 70–80% of all street-based IDUs in Sargodha, who were also demonstrating very little sharing on independent assessments.

## Methods

We interviewed approximately 150 IDUs in 4 focus group discussions and 30 outreach workers in one focus group discussion, in July 2007. Although their specific demographic data were not collected for this assessment, IDUs in Sargodha are a median of 30 years old with a range from 15 to 70 years. All participants were males and ethnic Punjabis. Since outreach workers are hired from IDUs, they have similar demographic characteristics. We conducted one discussion with IDUs in a rural location and two at urban locations, while the drop in center discussion had both urban and rural participants. These sites were selected randomly. IDUs were recruited on the basis of their presence at the injection sites when the study team arrived. All IDUs present at the site at the time were allowed to participate on a voluntary basis. None were excluded if they wanted to participate. During the discussion, IDUs moved in and out of discussions freely. The discussion with out-reach workers was conducted after the IDU discussions in order to verify findings and to add detail. All outreach workers working for the NGO in the city were specially asked to attend this discussion.

The discussions were guided by a pre-designed questionnaire that were pre-tested with a smaller group of IDUs at the local drop in center. The questionnaires asked about the type and frequency of drugs used, injecting practices, sharing behaviors and sexual practices. The discussants were encouraged to add details beyond specific questions, including topics that they felt were pertinent but had not been included in the questionnaire. The discussions were led by ABA who was assisted by AAK. The study team took notes of salient points but did not transcribe or record the discussions. The points were repeated back to the group during and at the end of the discussion to assure their agreement with the notes and to solicit further comments. No software or coding scheme were applied to data. Discussions were continued until saturation was reached for the themes that were emerging. The study team met at the end of each discussion to go over the answers received and to assure that our questions were sufficient to address any emerging themes. It was decided that pertinent emerging themes were reasonably covered by our questionnaire and by allowing participants to elaborate freely on the themes observed.

Routine assessments to inform about aspects of delivery of services are integral to our project design. These are conducted monthly by our city level team and quarterly or biannually by the national team. These frequently involve discussions with IDUs and outreach workers, and allows them to influence the services they receive or provide. As no personal information is sought or an individual identified, additional ethical review is not sought. The same principle applied to this assessment. Since the drug user interviews/discussions were held in open public spaces participants freely moved in and out of the group and were not coerced into answering our queries.

No compensation was provided to participants. The NGO routinely inquires about aspects of delivery of services from its clients. These services are considered sufficient compensation for our clients to for the time they spend with our staff. Finally, since this rapid assessment explored the reasons for rise in prevalence that appeared consistent across data sources (national surveillance and our own VCT), no additional biological tests were performed.

## Results

IDUs that participated in our discussions were all men, aged 15 to 70 years and ethnic Punjabis. Outreach workers are all former IDUs and therefore have a similar demographic profile. Some IDUs are small farmers or day-laborers, however, most IDUs claimed their income is from begging, odd jobs or garbage collection. We found a few affluent businessmen in the drop-in center group. Outreach workers describe considerable contribution from theft. Only 294 of our currently registered 1400 clients sleep on the street at night, the remaining return to their homes. Few (an estimated 5–6%) felt they were sexually active. We saw only one woman IDU (who was under the influence and did not participate in the discussion) at any of the locations. Women IDUs are uncommon on the street as described by other IDUs and peer workers. Most IDUs were local residents.

Six rural and 28 urban injecting locations (called "spots" locally) were identified in Sargodha. A typical urban location is situated in a large vacant lot in a residential neighborhood and has about 20–30 IDUs at any given time and about 100 daily. Rural locations are located in clearings in the middle of farms and can have about 30–40 IDUs at a time and about 100–200 daily. Shooting galleries have not been observed in Sargodha. Within each location, IDUs inject in subgroups that retain about the same members for days or weeks to some extent. While specific location of spots within neighborhoods change due to police raids or community pressure, the overall neighborhoods where injecting occurs remain the same over the years, i.e. when relocating, spots tend to stay in the same neighborhoods in the city. This is truer for urban locations than for rural ones. Injecting activities continue during all daylight hours at all spots.

Many IDUs move between spots on any given day, mostly to adjacent or nearby spots. Most return to their own spot either the same or the next day. Approximate motility of an IDU is about 1–2 spots per week. Longer-term mobility either between spots or to other cities is less common and is usually motivated by family relocations and infrequently due to drugs availability.

Some IDUs inject and smoke heroin on the same day. The commonest reasons for switching to injecting are financial. Injected drugs give a greater "high" and therefore are economical (most IDUs claim that it takes 2–3 times higher amounts of smoked heroin to produce the same level of "high"). Injecting drugs is relatively new for Sargodha and started around 10–12 years ago but has taken off in the recent years. We found that with the exception of a few IDUs that had injected for more than 10 years, most IDUs had started injecting within the past 2 years and nearly all for less than 5 years. Consequently, most were not yet proficient in injecting themselves and turned to street injectors (called "street doctors"). These are more experienced IDUs that inject others for a fee. Typically there were 2–3 injectors present at a spot of 30 IDUs and each injected about 15–16 persons daily.

Heroin is almost exclusively the drug used in Sargodha. It is available in single dose sachets (locally called a "token"). Most claim to inject 5–7 times a day. It is nearly always dissolved in Phenirimine (Avil^®^, an antihistamine) which is felt to augment the "high" of heroin. The most frequently used form of Phenirimine is a veterinary formulation, which is sold in multi-user vials that look like they had been refilled at home. Less of often (at one spot where Phenirimine was unavailable) lemon juice is used to dissolve heroin.

An injection costs about Rs 60–70 (USD 1–1.15) of which the drug is about Rs 40, the syringe: Rs 5, Phenirimine: Rs 5, and injection fee: Rs 10–20. Usually the group or the street injector will procure the Phenirimine multi-user vial (stated price: Rs 38, usual street value: Rs 10–20) which yields about 10 injections. The drugs and paraphernalia are paid for in cash, syringes or a share of the dissolved drug called "scale" (described below).

Nearly all IDUs claim to use fresh syringes all the time. However since they receive only 2 syringes a day, many reuse their own syringes. Some sell used syringes to others and to local pharmacies. Used syringes cost about the same as new ones.

Syringes are loaded, injected and shared by a locally developed method called "scale". The IDU procures and brings heroin powder to the injector who then dissolves it in Phenirimine making up a 5 ml mixture. This mixing can happen by either "back-loading" drug powder in the syringe (the plunger is removed, the drug powder is placed in the syringe and the plunger is replaced) and drawing up 5 ml of Phenirimine directly from the vial; or the dried drug powder and the Phenirimine are mixed together in a separate container, and then drawn up into the syringe. During drug injection, most Pakistani IDUs prefer to double pump the syringe, where the plunger is pushed half way through and then retracted (pulling back some blood) and then rapidly pushed to the desired amount. The injector typically injects 3 ml and keeps 2 ml for himself, which he will aliquot into his own new or old syringe. The injector's portion is called "scale". The injector usually pools all the leftover "scale" either for his own use or to sell it. "Scale" is worth slightly less than the regular drug as IDUs feel it is not as strong as the regular drug.

## Discussion

Use of an unprecedented method of injecting drugs that bypasses fresh syringes, larger sharing networks, higher injection frequency and common use of street injectors seem to explain the rapid rise in HIV prevalence among IDUs in Sargodha despite high level of provision of fresh syringes.

The method of sharing syringes called "scale" appears the main reason for the rapid rise in HIV in Sargodha. This process is multi-factorial. IDUs in Sargodha are poorer than those in other cities and start injecting about 2 years into their drug use rather than the 6–7 years it takes in other cities[6-8,24]. This time may be too short for many users to learn to inject themselves and many rely on street injectors. Fairbairn et al have described higher HIV risk associated with street injectors[25]. Poverty also limits the ability to pay for the drugs, paraphernalia and services; which are then paid for by sharing the drug by a method called "scale". The shared drug is mixed with blood due to the double pumping that appears to be common among Pakistani IDUs. This sharing method essentially bypasses fresh syringes as the drug-blood is passed on and injected with new syringes, in effect causing a near universal sharing of drug-blood mixture between IDUs, even when a new syringe is used. Since the "scaled" drug mixture is sold to be injected as is (or back-loaded into the IDU's own fresh syringe), an important protection – that of cleaning used syringes[26,27] – is also bypassed. Mixing of drugs prior to being aliquoted for multiple IDUs has been described previously from Hungary[28] and Russia (Arkadiuz Majszyk, personal communication). However, the double pumping of syringe with consequent mixing of blood with drug renders the Sargodha experience riskier. Finally, this practice is novel and was completely unforeseen by us and missed by the national surveillance which is quantitative. Our findings highlight the significance of qualitative assessments as routine parts of program evaluations.

Sargodha is on one of the main heroin trafficking routes from Afghanistan. Drug traffickers "off-load" some drug in Sargodha. Off-loading is the phenomenon where a certain amount of drug is sold off cheaply in local markets in order to meet local expenses. This partly explains why heroin found in Sargodha is relatively pure and cheaper. Heroin requires more frequent injections[29], in part due to its short half life of 9–22 minutes[30]. While IDUs from other cities use cocktails of synthetic opioids and benzodiazepines, and inject 2–3 times daily (national average: 2.7)[7,8], IDUs from Sargodha use heroin exclusively[8] and may inject 6–7 times daily, enhancing their potential exposure to blood from HIV-infected compatriots.

Size and density of sharing networks are key determinants of sexual transmission of HIV and STIs [31-35]. A similar dynamic may operate for syringe sharing networks, particular when large groups of IDUs sit together and inject, although this remains unproven. The syringe sharing networks in Sargodha are 2–3 folds larger than in most other Pakistani cities, due to unclear reasons. These larger networks may have contributed to the rapid rise in HIV prevalence. Our observations warrant more detailed study of how these networks form and operate and why they are larger in Sargodha than elsewhere.

There are several limitations of this assessment. This is a brief assessment of situation and did not use a scientifically rigorous sampling frame. However, we discussed with nearly 200 current or recent former IDUs in a city with about 2500 IDUs. Even if the sampling frame was not fully representative, our study sampled the concerns of a large proportion of the IDUs in the city. Secondly, this is the first study in Pakistan that suggests that large groups and networks of IDUs may play a role in rapid transmission of HIV among IDUs in the country. However, we did not study the network structure of the injecting community. Our recruitment process may have selected for IDUs that were staying at the injecting sites after their injections and were therefore more "hardcore" IDUs. IDUs that had just arrived at the site seeking drug were more likely to have been in withdrawal and those that had just received their injection were more likely to be in stupor. Both were less likely to participate in our discussions. This may have accounted for the higher injection frequency than was reported by the national surveillance which took a more probability sample of all IDUs. Finally we focused only mainly on injecting rather than sexual behaviors, since very few IDUs claimed to be sexually active. In the national surveillance, 46% of IDUs had reported some sexual activity in the past 6 months[5], however in another study (Ahmad et al, under review), this amounted to about one sex act per month. Sex between IDUs is also not well studied in Pakistan and may have been important in the rapid HIV transmission that was observed in the city.

Several interventions are necessary to reduce HIV transmission in Sargodha. We have already increased the supply of syringes to match the injecting frequency in Sargodha and have started supplying single user vials of Phenirimine and mixing utensils. We are now designing behavior change interventions for street injectors and will hire them as peer workers or will pay them to not mix or sell "scale".

Our study was a quick assessment to understand the explosive rise in HIV in one city. We found an unprecedented method of sharing syringes, more frequent injections and large sharing networks of IDUs. Such variations in patterns from other cities underscore the importance of understanding and using local context to guide harm reduction programs. We also highlighted the importance of ongoing research to inform program implementation.

## Competing interests

The authors declare that they have no competing interests.

## Authors' contributions

AAK conceived the study, conducted field interviews, collated data, analyzed the data and drafted the manuscript. ABA helped in the study design and the development of the questionnaires, conducted field interviews and contributed to the manuscript. SUHQ helped with the study design, helped develop the questionnaires, conducted field interviews and contributed to the manuscript. AR helped conceive the study and contributed to the manuscript. STZ helped conceive the study, participated in analysis and contributed to the manuscript.

All authors have read and approved the final manuscript.
